# Bis(catecholato-κ^2^ *O*,*O*′)bis­(dimethyl sulfoxide-κ*O*)titanium(IV)

**DOI:** 10.1107/S2056989022002638

**Published:** 2022-03-15

**Authors:** Nisansala Hewage, Carolyn Mastriano, Christian Brückner, Matthias Zeller

**Affiliations:** a Department of Chemistry, University of Connecticut, Storrs, CT 06269-3060, USA; b Department of Chemistry, Purdue University, 560 Oval Drive, West Lafayette, IN, 47907-2084, USA

**Keywords:** transition-metal catecholates, heteroleptic catecholates, titanium catecholates, crystal structure

## Abstract

Bis(benzene-1,2-diolato-κ^2^ *O*,*O*′)bis­(dimethyl sulfoxide-κ*O*)titanium(IV), [Ti(C_6_H_4_O_2_)_2_(C_2_H_6_OS)_2_], crystallizes with two crystallographically independent mol­ecules in the space group *P*2_1_/*c* emulating ortho­rhom­bic *Pbca* symmetry (β = 90.0445 (9)°]. The two mol­ecules are related by pseudo-glide symmetry, broken by modulation of each one catecholate and dimethyl sulfoxide (DMSO) ligand. Twinning by pseudomerohedry was observed [twin ratio 0.5499 (7):0.4401 (7)]. Complex **3** was obtained by heating of diprotonated titanium tris-catecholate precursor **2^H^** in DMSO, by formal displacement of a catechol mol­ecule by two DMSO mol­ecules. Complex **3** is just the second heteroleptic, mono-nuclear, neutral bis-catecholate complex with TiO_6_ metal coordination, the only other one being its bis-DMF analogue **6**. The two mol­ecules of **3** exhibit a distorted octa­hedral geometry. The geometry and distortions from ideal symmetry of **3** are discussed and compared to **6** and to cationic tris-catecholate titanium complexes.

## Abstract 2

Bis(catecholato)bis­(DMSO)titanium crystallizes with two crystallographically independent mol­ecules related by pseudo-glide symmetry in a distorted octa­hedral O_6_ donor-atom geometry around titanium.

## Chemical context

**Figure 1:**
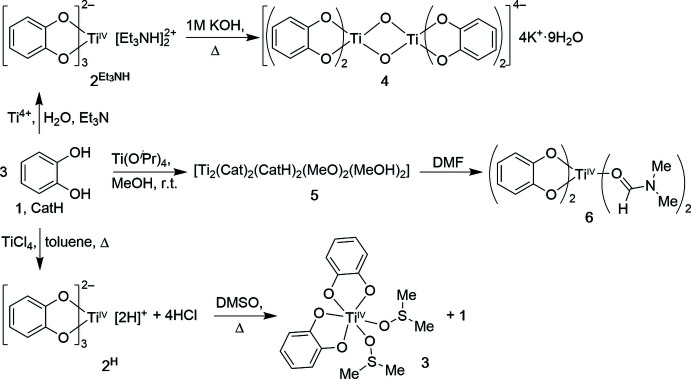
Formation of titanium(IV) catecholate complexes, including the title compound **3**.

The dianion of catechol (1,2-di­hydroxy­benzene, CatH, **1**) is a bidentate, dianionic and non-innocent *O,O-*chelating agent with a particularly high affinity for HSAB hard-metal ions, *i.e*., ions of high oxidation states or high charge-to-metal-ion-radius ratios (Pierpont & Lange, 1994 ▸; Kaim & Schwederski, 2010 ▸). Titanium(IV) is one such metal ion and long known to form stable, pseudo­octa­hedral tris­catecholate complexes, such as **2^Et3NH^**, by reaction of catechol with Ti^4+^ sources under basic conditions (Fig. 1 ▸) (Borgias *et al.*, 1984 ▸).

Titanium catecholate complexes have found various uses: Titanium tris­catecholate complexes of the alkaline earth metals were utilized as mol­ecular precursors to a number of *M* ^II^TiO_6_-type perovskites (Ali & Milne, 1987 ▸; Marteel-Parrish *et al.*, 2008 ▸). Titanium catecholates have been exploited as catalysts in acetyl­ene hydrogenation (Bazhenova *et al.*, 2016 ▸) while three-dimensional titanium catecholate frameworks of high proton conductivity (Nguyen *et al.*, 2015 ▸) and titanium catecholate-based MOFs have been described (Cao *et al.*, 2020 ▸). Metal catecholates have been suggested as adsorbents for toxic gases (Bobbitt & Snurr, 2018 ▸). Titanium tris­catecholates were also used to self-assemble a potential bimodal contrast agent (Dehaen *et al.*, 2012 ▸). A number of heteroleptic mono- and multi-nuclear titanium complexes do contain titanium catechol units (Sakata *et al.*, 2010 ▸; Bazhenova *et al.*, 2016 ▸; Sonström *et al.*, 2019 ▸; Passadis *et al.*, 2020 ▸) (for further examples, see *Database survey* below). Most prominently, titanium tris-catecholates have been used as versatile building blocks in a range of supra­molecular, oligonuclear homo- and hetero-metal-ligand cluster assemblies (Brückner *et al.*, 1998 ▸; Caulder *et al.*, 2001 ▸; Albrecht *et al.*, 2008 ▸, 2019 ▸).

**Figure 2:**
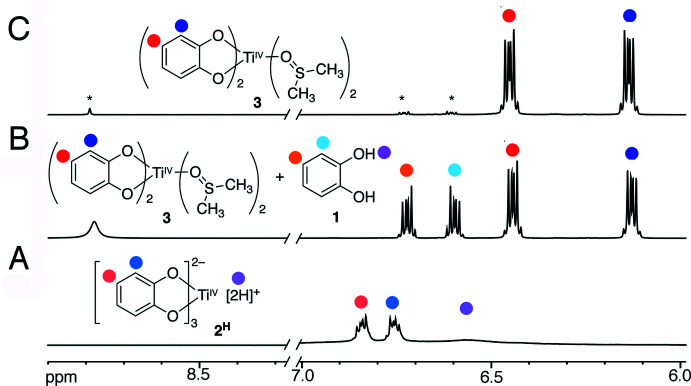
^1^H NMR spectra (400 MHz, DMSO-*d*_6_) of (*A*) complex **2^H^** dissolved at ambient temperature; (*B*) of complex **2^H^** at ∼373 K, showing the presence of complex **3** and free catechol **1**; (*C*) of isolated crystals of **3** precipitated from DMSO at ambient temperature (* indicates the presence of residual **1**).

Reaction of TiCl_4_ under anhydrous conditions in toluene generates a brick-colored amorphous powder of the diproton­ated titanium tris-catecholate complex **2^H^** (Davies & Dutremez, 1990 ▸). We found that this compound dissolves sufficiently enough in ambient-temperature DMSO-*d*_6_ to record a simple ^1^H NMR spectrum, showing only two multiplets of equal integration (at 6.84 and 6.75 ppm), corres­ponding to the *ortho*- and *meta*-hydrogens on three near-identical catecholate moieties (the counter-cations – protons – are believed to be dynamically associated with the trigonal faces of the pseudo-octa­hedral coordination sphere formed by the six catecholate oxygens) (Fig. 2 ▸ *A*). Upon heating **2^H^** in DMSO (or DMSO-*d*_6_), its solubility increases drastically. When followed by ^1^H NMR spectroscopy, the formation of a new species with two catecholate signals (*m* at 6.42 and 6.12 ppm, in 2:2 intensity) and 1 equivalent of free catechol (*m* at 6.73 and 6.60, *br s* at 8.1 ppm, all 1:1:1) can be observed (Fig. 2 ▸ *B*). Upon cooling, dark red–orange crystals of the title compound **3** formed. Isolated and analyzed by NMR spectroscopy, they exhibit only the signals for the new species formed (Fig. 2 ▸ *C*) and the signals for two slightly high-field-shifted DMSO mol­ecules (not shown).

**Figure 3:**
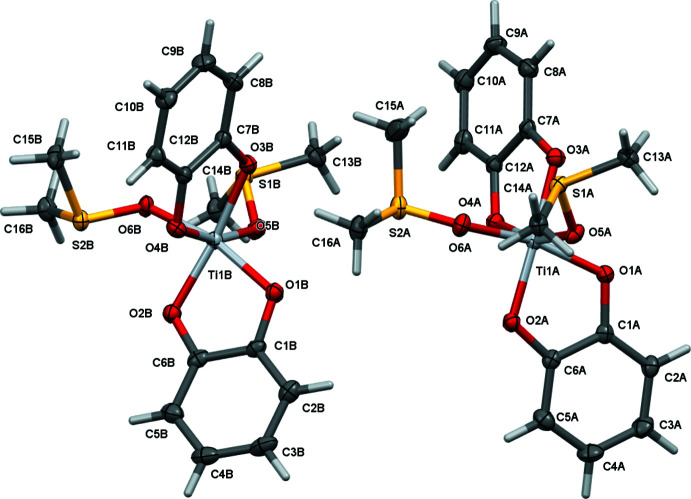
The two crystallographically independent mol­ecules of **3**. View along the *a* axis. Mol­ecules *A* and *B* are related by pseudo-glide operations (see discussion for details).

The material was also analyzed by single crystal X-ray diffraction (Fig. 3 ▸). Evidently, one protonated catecholate ligand (*i.e*., catechol **1**) of the starting tris­catcholate complex **2^H^** was exchanged for two DMSO mol­ecules, coordinating through their oxygen atoms in adjacent positions, thus forming a neutral, heteroleptic, mononuclear octa­hedral complex. Details of the structural arrangement will be discussed in the *Structural commentary* section below. 
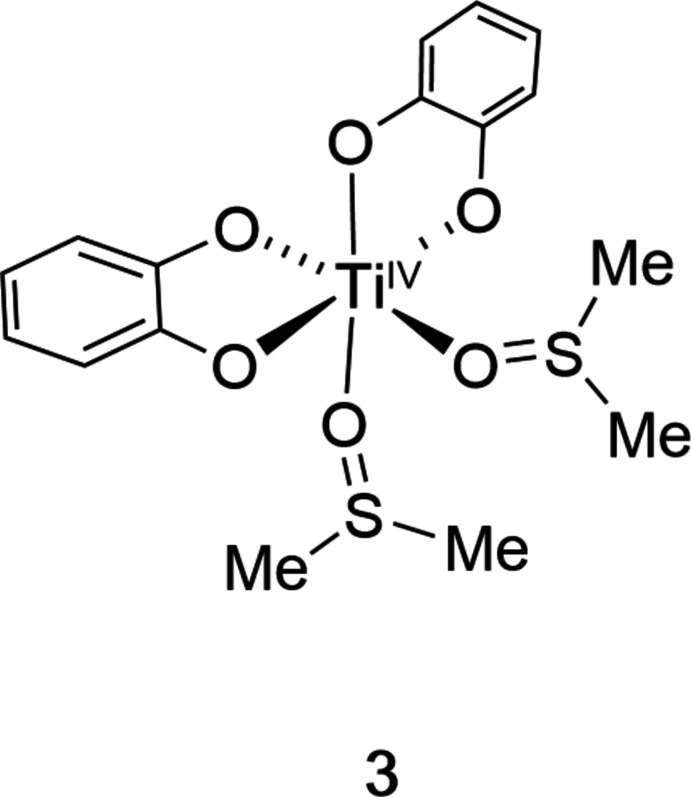


**Figure 4:**
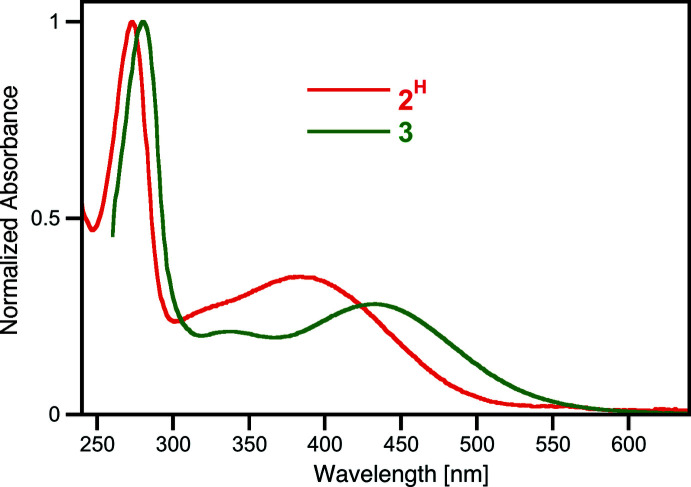
Normalized UV–vis spectrum of title compound **3** (DMSO) in comparison to that of the starting tris­catecholate **1** (H_2_O).

The UV–vis spectrum of the orange solution of **3** is overall similar to that of the starting material **2^H^**; both spectra are dominated in the visible range by broad, little-structured catecholate ligand-to-metal charge-transfer bands (for **3**, *l*_max_ = 441 nm; half-height width > 150 nm; Fig. 4 ▸). In comparison to the spectrum of **2^H^**, all bands for **3** are bathochromically shifted.

## Structural commentary

The title complex **3**, having solution *C*_2_ symmetry, crystallizes as a racemic mixture with two crystallographically independent mol­ecules in the monoclinic space group *P*2_1_/*c* (Fig. 3 ▸). For both mol­ecules, the solution *C*_2_ symmetry is broken in the solid state, and the Ti atoms are each bonded to two chelating catecholate and two monodentate O-coordinated di­methyl­sulfoxide ligands. The Ti—O_Cat_ bond distances range from 1.9113 (19) to 1.9564 (18) Å in mol­ecule *A*, and 1.9108 (18) to 1.9545 (18) in mol­ecule *B* [average 1.93 (3) Å]. A notable structural *trans*-effect is observed as the longer distances are observed for the oxygen atoms opposite another catechol oxygen donor atom [1.9346 (18) to 1.9564 (18) Å] while the shorter distances [1.9108 (18) to 1.9284 (19) Å] are found *trans* to the weaker electron-donating O_DMSO_ atoms.

The Ti—O_Cat_ bond distances in the cationic tris­catecholate complex **2^Et3NH^** are on average longer [1.97 (3) *vs* 1.93 (3) Å in **3**], ranging from 1.941 (1) to 2.014 (1) Å with differences between long and short Ti—O bonds caused by distortion from strong hydrogen bonds to the Et_3_NH^+^ counter-cations (reflected in a 0.05 Å lengthening of the associated Ti—O bonds) (Borgias *et al.*, 1984 ▸). The four Ti—O_DMSO_ bond distances in **3** are at 2.0214 (19) to 2.0416 (18) Å significantly longer than the Ti—O_Cat_ bond lengths, as would be expected for neutral and uncharged DMSO ligands.

The bond lengths in **3** also compare well with those of the [bis­catecholate-bis-DMF]titanium complex **6**, the DMF analogue to the title compound (Bazhenova *et al.*, 2016 ▸) and the only other reported heteroleptic mono-nuclear and uncharged bis-catecholate titanium complex with *M*O_6_ metal coordination (see *Database survey*). The Ti—O_cat_ bond lengths in **6** are 1.9003 (12) and 1.9181 (12) Å for the oxygen atoms *trans* to the DMF mol­ecules, 1.9408 (11) and 1.9483 (11) Å when *trans* to another O_cat_ atom, and 2.0396 (12) and 2.0736 (12) Å for the Ti—O_DMF_ bond lengths. They thus closely mirror those found in **3**.

The small bite angles of the chelating catecholate anions induce substantial distortions from idealized octa­hedral symmetry. The catecholate O—Ti—O angles in **3** are 80.73 (7) and 81.00 (8)° in mol­ecule *A* and 80.85 (8) and 80.24 (8)° in mol­ecule *B*, which are essentially indistinguishable from those in **2^Et3NH^** [80.1 (1) to 80.6 (1)°] and **6** [O3 80.68 (5) and O2 80.97 (5)°]. The other *cis* angles in **3** cover a wide range from as small as 82.35 (7)° (for the O_DMSO_—Ti—O_DMSO_ angle in mol­ecule *A*) to as large as 105.30 (8)° (for one of the O_cat_—Ti—O_DMSO_ angles in mol­ecule *B*). The latter rather obtuse large angle is unique in being nearly 4° wider than the next largest angle [its equivalent in mol­ecule *A* is 101.66 (8)°]. The angles in **2^Et3NH^** do not exceed 101.3°. The equivalent angles in the DMF analogue **6** are more evenly distributed than in either mol­ecule of **3**, ranging from 82.53 (5) to 97.77 (5)°.

This more pronounced deviation from ideal octa­hedral symmetry for mol­ecule *B* of **3** is also confirmed by a more holistic analysis, using a normalized root-mean-square deviation algorithm to calculate the distortion from octa­hedral symmetry as implemented in the program *SHAPE* (Pinsky & Avnir, 1998 ▸; Alvarez *et al.*, 2002 ▸; Casanova *et al.*, 2004 ▸). The calculated continuous shape measures (CShM’s) relative to ideal reference octa­hedral symmetry are 1.434 for **2^Et3NH^**, 1.513 for **6**, 1.491 for less distorted mol­ecule *A* of **3**, and 1.854 for mol­ecule *B* (Table 1 ▸). Shape measures may be between 0 and 100 where zero represents a perfect fit for the selected shape, and CShM values of less than 1.0 are usually inter­preted as only minor distortions from the reference shape. Values between 1 and 3 indicate substantial distortions, but the reference shape still provides a good stereochemical description (Cirera *et al.*, 2005 ▸). For the four cases analyzed here, the CShM’s for the next best fit, trigonal prismatic, are all around 10 (Table 1 ▸). The geometries of **3**, **6** and **2^Et3NH^** are thus best described as distorted octa­hedral, being far removed from fitting any other polygon.

The two independent mol­ecules in compound **3** are related to each other by crystallographic pseudosymmetry. Complex **3** crystallized in a pseudo-ortho­rhom­bic setting with a refined β angle of 90.0445 (9)°, and emulates space group *Pbca* with additional *b*- and *a*-glide operations along the *a*- and *c*-axis directions. Exact translational symmetry is broken by modulation of one of the catecholate and one of the DMSO ligands, as discussed below. The metric pseudosymmetry allows for the possibility of twinning. Indeed, the crystal investigated was found to be pseudo-merohedrically twinned by symmetry elements of the emulated ortho­rhom­bic symmetry. Application of the twin transformation matrix 1 0 0, 0 −1 0, 0 0 −1 yielded close to equal twin components with a refined twin ratio of 0.5499 (7) to 0.4401 (7).

**Figure 5:**
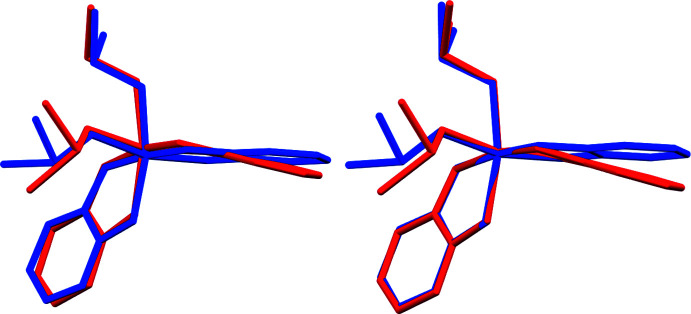
Root-mean-square overlays of the two independent mol­ecules of **3**, after inversion of mol­ecule *B* (red: mol­ecule *A*; blue: mol­ecule *B*). Left: fit based on all non-H atoms (r.m.s. deviation 0.459 Å). Right: fit based on Ti and O atoms only (r.m.s. deviation 0.056 Å).

A root-mean-square overlay of the two mol­ecules yields an r.m.s. deviation of 0.459 Å, indicating substantial variation between the geometries of mol­ecules *A* and *B* (Fig. 5 ▸). A similar overlay based on only the titanium and oxygen atoms gives a much smaller value of only 0.056 Å, indicating that the main differences between the two complexes is rooted in the ligands, even though there are small and noticeable differences for the TiO_6_ cores as well (with mol­ecule *B* deviating more from ideal octa­hedral symmetry than mol­ecule *A*, as discussed above). The main distinction between the two mol­ecules is, however, associated with substantial twists and torsions of the catecholate and DMSO ligands. The r.m.s. overlay reveals a close match of one of the catecholate ligands and one of the DMSO ligands. The other catecholate and DMSO ligands, on the other hand, do show substantial variation between the two mol­ecules. The catecholate of C7–C12 undergoes a twist-motion by a slight rotation around the O_cat_—O_cat_ axis. In mol­ecule *A* (blue in the overlay), the catecholate ligand is close to coplanar with the titanium atom, while in mol­ecule *B* (red in the overlay) the catecholate and the TiO(Cat)_2_ plane are clearly angled against each other. The deviations of the Ti atoms from the mean catecholate planes are 0.049 (2) and 0.349 (2) Å for mol­ecules *A* and *B*, respectively. The angle between the mean catecholate and Ti(OCat)_2_ planes is 1.97 (9)° for complex *A*, but a much larger value of 13.6 (8)° for complex *B*.

The other main difference between the two mol­ecules is a rotation of about 14° for one of the two DMSO ligands around the Ti—O bond, which can be expressed *via* the torsion angle O5—Ti1—O6—S2 (S2 is the sulfur atom of the rotated DMSO ligand, O5 the oxygen atom of the other invariant DMSO ligand). These torsion angles are 165.13 (16)° for mol­ecule *A*, and 151.10 (14)° for mol­ecule *B*. The largest overall motion is observed for the DMSO methyl groups of C15 [1.774 (3) Å in the r.m.s. overlay based on the titanium and oxygen atoms].

**Figure 6:**
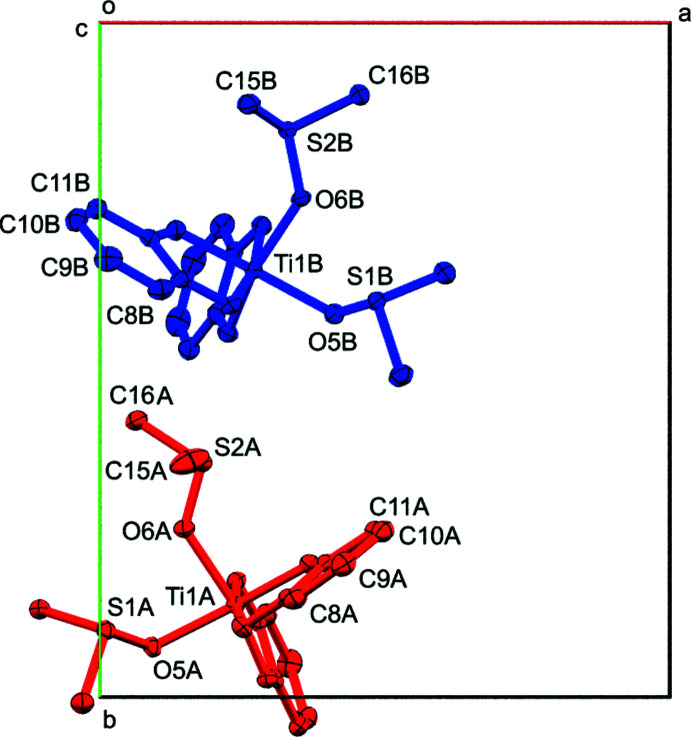
View of **3** down the *c* axis, showing the modulation by pseudo-*Pbca* symmetry. Mol­ecules color coded by symmetry equivalence (red: mol­ecule *A*, blue mol­ecule *B*). Atom labels included for Ti, DMSO S and O atoms, and for C atoms with the largest modulation. 50% probability ellipsoids.

The differences in mol­ecular geometry between mol­ecules *A* and *B* and the modulation by pseudo-ortho­rhom­bic symmetry are closely related, showing mol­ecules *A* and *B* as they are related to each other by a pseudo *b*-glide perpendicular to (100) (Fig. 6 ▸). In addition to the variations in mol­ecular geometry seen in the mol­ecule overlay, a very slight rotation of the entire complex is also observed.

## Supra­molecular features

The most prominent directional inter­actions in complex **3** are medium strength C—H⋯O inter­actions, involving the DMSO methyl groups as hydrogen-bond donors, and catecholate and DMSO oxygen atoms as the respective acceptors. Hydrogen bonds with C⋯O distances below 3.50 Å are given in Table 2 ▸. Some of these H⋯O distances are unusually short for C—H⋯O inter­actions, with H⋯O distances as short as 2.27 and 2.34 Å, approaching distances usually only observed for classical hydrogen bonds involving acidic hydrogens. This might indicate stronger than usual inter­actions with a possibly larger influence on the packing and mol­ecular arrangement in the solid state than usually observed for C—H⋯O inter­actions.

**Figure 7:**
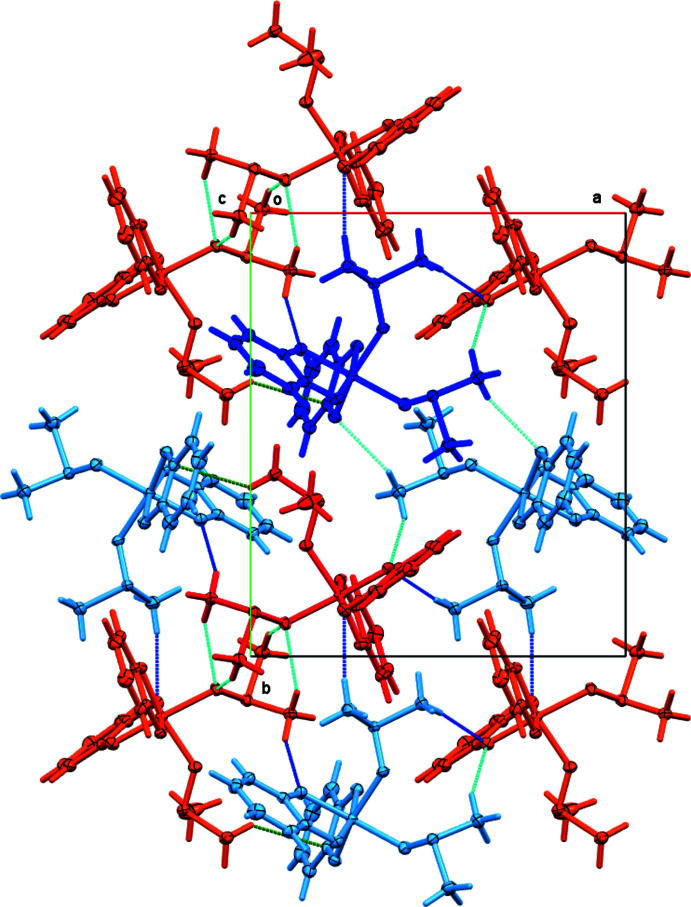
Directional inter­actions in **3**, viewed down the *c* axis. Red: mol­ecule *A*, blue: mol­ecule *B*. Lighter colored mol­ecules are generated by crystal symmetry. Dark-blue dashed lines: C—H⋯O bonds with H⋯O distance < 2.5 Å; light-blue dashed lines: C—H⋯O bonds with H⋯O distance between 2.5 and 2.62 A; green dashed lines C—H⋯π contacts. 50% probability ellipsoids.

When plotting the C—H⋯O hydrogen bonds (Fig. 7 ▸), it becomes evident that the inter­actions are different for the two mol­ecules, despite their close relationship by a pseudo-glide operation. Inter­actions involving the less modulated fragments of **3A** and **3B** exhibit similar hydrogen-bonding environments. C13 and C14 of the less-modulated DMSO mol­ecule exhibit the same type of hydrogen bonds to O1, O4 and O5 in neighboring mol­ecules (see Table 2 ▸ for symmetry operators and numerical values). The exact bond lengths for C14 vary slightly, a bond to O5 is broken and one to O4 significantly elongated for mol­ecule *B*, but the overall hydrogen-bonding pattern for this DMSO fragment is very similar for both mol­ecules *A* and *B*. This is not the case for the other significantly modulated DMSO mol­ecule. For **3B**, three significant C—H⋯O inter­actions are observed, towards O1*A*, O3*A* and O4*A* of neighboring entities. None of these are found for **3A**. All hydrogen-to-oxygen distances are beyond what could be still regarded as attractive and stabilizing. Methyl carbon atom C16*A* is at a distance of 3.128 Å from O1*B*, close enough for a C—H⋯O hydrogen bond to be suspected, but its hydrogen atoms are rotated such that the H⋯O distances are > 2.8 Å, and the C—H⋯O angles are unfavorable at 97.9 and 99.5° (H-atom positions were clearly resolved in difference-density maps and were allowed to rotate to fit the experimental electron density). The shortest distance involving the H atoms of C16*A* is instead towards C1*B* of a neighboring catecholate ring (2.734 Å, shown as a green dashed line in Fig. 7 ▸), and C15*A* does not exhibit any H⋯*X* contacts < 2.8 Å. This clear difference between the hydrogen-bonding inter­actions of C15 and C16 in the two mol­ecules is clearly related to the modulation that breaks the exact *Pbca* glide symmetry in the structure of **3**. It is not clear whether the ability to form stronger inter­actions is the cause for the modulation, or whether the modulation causes the differences in inter­molecular inter­actions and the modulation itself is caused by other less-directional forces such as dispersive inter­actions. Most likely the concerted effects of both modulation and inter­molecular inter­actions reinforcing and stabilizing each other lead to the observed packing of the mol­ecules.

## Database survey

A database survey of titanium catecholate complexes reveals a plethora of homoleptic tris­catecholates but only a few in monometallic assemblies. A search of the Cambridge Structural Database (CSD, Version 5.42, accessed Feb 2021; Groom *et al.*, 2016 ▸) yields, in addition to **2^Et3NH^** (Borgias *et al.*, 1984 ▸), eleven other monocationic homoleptic tris­catecholate titanium complexes with only one metal center: SUKNUK (Kwamen *et al.*, 2020 ▸), SUKQAT (Kwamen *et al.*, 2020 ▸), GOJMIC (Tinoco *et al.*, 2008 ▸), LEXQUD (Dong *et al.*, 2018 ▸), LEXRAK (Dong *et al.*, 2018 ▸), MAGLAK (Van Craen *et al.*, 2016 ▸), VEPJUW (Davis *et al.*, 2006 ▸), VEPKAD (Davis *et al.*, 2006 ▸), VILXIX (Hahn *et al.*, 1991 ▸), XIKLOV (Chen *et al.*, 2018 ▸), and YUPNEF (Johnson *et al.*, 2020 ▸).

The conversion of tris­catecholate complexes to heteroleptic complexes has been observed previously, as the hydroxide-induced displacement of a catecholate ligand from **2^Et3NH^** to form the bis-(μ-oxo-bridged) bis­catecholate **4** exemplifies (Borgias *et al.*, 1984 ▸). Direct syntheses are also known (Sakata *et al.*, 2010 ▸). For example, treatment of titanium methoxide with a methano­lic solution of catechol **1** under ambient conditions resulted in the formation of a dinuclear heteroleptic complex **5** with a mixture of catechol/catecholate and methanol/methano­late ligands (Bazhenova *et al.*, 2016 ▸). Notably both examples of these heteroleptic complexes are multinuclear. Complex **5** dissolved in DMF, however, and exchanges all methanol/methano­lates for DMF and rearranges to form the mononuclear [bis­catecholate-bis-DMF]titanium complex **6**, the DMF analogue of the title compound (CCDC 1489371; Bazhenova *et al.*, 2016 ▸). Neutral and monometallic complexes of this kind are exceedingly rare. A search of the CSD yielded complex **6** as the only other heteroleptic mono-nuclear, neutral bis-catecholate complex with TiO_6_ metal coordination; complex **3** is only the second such complex.

## Synthesis and crystallization

Triscatcholate **2^H^** (500 mg, 1.34 × 10 ^−3^ mol), prepared as described in the literature (Davies & Dutremez, 1990 ▸), was dissolved at ∼173 K, in the minimal amount of DMSO (∼15 ml). The deep, dark-orange solution was allowed to cool slowly (in the water bath) to ambient temperature. The crystal mass that formed was broken up, placed on a porcelain frit, washed with minimal amount of cool DMSO (m.p. 292 K!), then cold diethyl ether, and dried under suction. The red–orange matted plates of **3** (320 mg, 0.76 × 10 ^−3^ mol, 57% yield) were analytically pure. By NMR spectroscopy (*cf*. Fig. 3 ▸ *B*), the reaction is qu­anti­tative; nonetheless, no attempt was made to increase the yield by recovery of more product from the filtrate. C_16_H_20_O_6_S_2_Ti (*M*_W_ = 420.32 g mol^−1^); ^1^H NMR (400 MHz, DMSO-*d*_6_): δ 6.45 (*m*, 2H, 4,5-CH), 6.13 (*m*, 2H, 3,6-CH), 2.31 (*s*, 3H, CH_3_) ppm; UV–vis (DMSO): λ_max_ (ɛ/*M* ^−1^ cm^−1^) = 280 (9000), 343 (2000), 441 (2500).

## Refinement

Crystal data, data collection and structure refinement details are summarized in Table 3 ▸. The structure exhibits pseudo-ortho­rhom­bic symmetry (*Pbca*) and is twinned by a 180° rotation around the *a-* or *c*-axis. Application of the transformation matrix 1 0 0, 0 − 1 0, 0 0 − 1 yielded a twin ratio of 0.5399 (7):0.4401 (7). The pseudo-ortho­rhom­bic symmetry is broken by modulation of one of the catecholate rings by up to 1.4 Å, and one of the DMSO ligands by over 1.7 Å.

C—H bond distances were constrained to 0.95 Å for aromatic C—H and to 0.98 Å for aliphatic CH_3_ moieties, respectively. *U*_iso_(H) values were set to a multiple of *U*_eq_(C) with 1.5 for CH_3_ and 1.2 for C—H units. Reflections 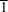 12, 112 and 013 were affected by the beam stop and were omitted from the refinement.

## Supplementary Material

Crystal structure: contains datablock(s) I. DOI: 10.1107/S2056989022002638/jy2018sup1.cif

Structure factors: contains datablock(s) I. DOI: 10.1107/S2056989022002638/jy2018Isup2.hkl

CCDC reference: 2157170

Additional supporting information:  crystallographic information; 3D view; checkCIF report

## Figures and Tables

**Table 1:** Continuous shape measures (CShM’s) relative to ideal reference octa­hedral symmetry for **2^Et3NH^**, **3** and **6**

Structure	Hexagon	Penta­gonal pyramid	Octa­hedron	Trigonal prism	Johnson penta­gonal pyramid J2
**2^Et3NH^**	33.773	23.482	1.434	9.808	27.215
**3A**	32.792	22.191	1.491	10.655	26.183
**3B**	32.830	21.007	1.854	10.159	24.952
**6**	33.664	22.467	1.513	10.069	26.511

**Table 2:** Hydrogen-bond geometry (Å, °)

*D*—H⋯*A*	*D*—H	H⋯*A*	*D*⋯*A*	*D*—H⋯*A*
C11*A*—H11*A*⋯O2*B* ^i^	0.95	2.62	3.491 (3)	153
C13*A*—H13*C*⋯O5*A* ^ii^	0.98	2.54	3.428 (3)	151
C14*A*—H14*B*⋯O1*A* ^ii^	0.98	2.66	3.378 (3)	130
C14*A*—H14*B*⋯O5*A* ^ii^	0.98	2.55	3.447 (3)	152
C14*A*—H14*C*⋯O4*B* ^iii^	0.98	2.27	3.193 (3)	156
C13*B*—H13*E*⋯O5*B* ^i^	0.98	2.60	3.495 (4)	151
C14*B*—H14*E*⋯O4*A* ^i^	0.98	2.55	3.438 (3)	151
C14*B*—H14*F*⋯O1*B* ^i^	0.98	2.56	3.336 (3)	136
C15*B*—H15*D*⋯O3*A* ^iv^	0.98	2.34	3.307 (3)	171
C16*B*—H16*D*⋯O1*A* ^iv^	0.98	2.59	3.186 (3)	119
C16*B*—H16*E*⋯O4*A* ^i^	0.98	2.45	3.427 (3)	173

Symmetry codes: (i) 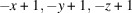 ; (ii) 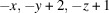 ; (iii) 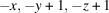 ; (iv) 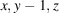 .

**Table 3:** Experimental details

Crystal data	
Chemical formula	[Ti(C_6_H_4_O_2_)_2_(C_2_H_6_OS)_2_]
*M*_r_	420.34
Crystal system, space group	Monoclinic, *P*2_1_/*c*
Temperature (K)	100
*a*, *b*, *c* (Å)	12.4531 (3), 14.7287 (3), 20.3676 (5)
β (°)	90.0445 (9)
*V* (Å^3^)	3735.78 (15)
*Z*	8
Radiation type	Mo *K*α
μ (mm^−1^)	0.71
Crystal size (mm)	0.35 × 0.23 × 0.09

Data collection	
Diffractometer	Nonius Kappa CCD
Absorption correction	Multi-scan (*SCALEPACK*; Otwinowski & Minor, 1997 ▸)
*T*_min_, *T*_max_	0.668, 0.939
No. of measured, independent and observed [*I* > 2σ(*I*)] reflections	39962, 8552, 7393
*R*_int_	0.047
(sin θ/λ)_max_ (Å^−1^)	0.666

Refinement	
*R*[*F* ^2^ > 2σ(*F* ^2^)], *wR*(*F* ^2^), *S*	0.036, 0.073, 1.04
No. of reflections	8552
No. of parameters	461
H-atom treatment	H-atom parameters constrained
Δρ_max_, Δρ_min_ (e Å^−3^)	0.41, −0.45

Computer programs: *COLLECT* (Nonius, 1998 ▸), *HKL-3000* (Otwinowski & Minor, 1997 ▸), *SHELXS97* (Sheldrick, 2008 ▸), *SHELXL2018/3* (Sheldrick, 2015 ▸), *ShelXle* (Hübschle *et al.*, 2011 ▸), *Mercury* (Macrae *et al.*, 2020 ▸), and *publCIF* (Westrip, 2010 ▸).

## supplementary crystallographic information

### Crystal data

**Table:**

[Ti(C_6_H_4_O_2_)_2_(C_2_H_6_OS)_2_]	*F*(000) = 1744
*M**_r_* = 420.34	*D*_x_ = 1.495 Mg m^−^^3^
Monoclinic, *P*2_1_/*c*	Mo *K*α radiation, λ = 0.71073 Å
*a* = 12.4531 (3) Å	Cell parameters from 39962 reflections
*b* = 14.7287 (3) Å	θ = 1.4–28.3°
*c* = 20.3676 (5) Å	µ = 0.71 mm^−^^1^
β = 90.0445 (9)°	*T* = 100 K
*V* = 3735.78 (15) Å^3^	Plate, orange
*Z* = 8	0.35 × 0.23 × 0.09 mm

### Data collection

**Table:**

Nonius Kappa CCD diffractometer	8552 independent reflections
Radiation source: fine focus X-ray tube	7393 reflections with *I* > 2σ(*I*)
Graphite monochromator	*R*_int_ = 0.047
ω and φ scans	θ_max_ = 28.3°, θ_min_ = 1.4°
Absorption correction: multi-scan (SCALEPACK; Otwinowski & Minor, 1997)	*h* = −16→14
*T*_min_ = 0.668, *T*_max_ = 0.939	*k* = −19→18
39962 measured reflections	*l* = −25→27

### Refinement

**Table:**

Refinement on *F*^2^	Secondary atom site location: difference Fourier map
Least-squares matrix: full	Hydrogen site location: inferred from neighbouring sites
*R*[*F*^2^ > 2σ(*F*^2^)] = 0.036	H-atom parameters constrained
*wR*(*F*^2^) = 0.073	*w* = 1/[σ^2^(*F*_o_^2^) + (0.0246*P*)^2^ + 2.3086*P*] where *P* = (*F*_o_^2^ + 2*F*_c_^2^)/3
*S* = 1.04	(Δ/σ)_max_ = 0.001
8552 reflections	Δρ_max_ = 0.41 e Å^−^^3^
461 parameters	Δρ_min_ = −0.45 e Å^−^^3^
0 restraints	Extinction correction: SHELXL2018/3 (Sheldrick 2015), Fc^*^=kFc[1+0.001xFc^2^λ^3^/sin(2θ)]^-1/4^
Primary atom site location: structure-invariant direct methods	Extinction coefficient: 0.00192 (16)

### Special details

**Table:**

Geometry. All esds (except the esd in the dihedral angle between two l.s. planes) are estimated using the full covariance matrix. The cell esds are taken into account individually in the estimation of esds in distances, angles and torsion angles; correlations between esds in cell parameters are only used when they are defined by crystal symmetry. An approximate (isotropic) treatment of cell esds is used for estimating esds involving l.s. planes.
Refinement. The structure exhibits pseudo-orthorhombic symmetry (Pbca) and is twinned by a 180 degree rotation around the a or c-axis. Application of the twin matrix 1 0 0, 0 -1 0, 0 0 -1 yielded a twin ratio of 0.5399 (7) to 0.4401 (7). The pseudo-orthorhombic symmetry is broken by modulation of the phenylene rings by up to 1.4 Angstrom.

### Fractional atomic coordinates and isotropic or equivalent isotropic displacement parameters (Å^2^)

**Table:**

	*x*	*y*	*z*	*U*_iso_*/*U*_eq_	
Ti1A	0.23792 (4)	0.86347 (3)	0.47159 (2)	0.01639 (11)	
S1A	0.01041 (6)	0.90084 (4)	0.40944 (3)	0.01861 (14)	
S2A	0.17900 (6)	0.65300 (4)	0.43411 (4)	0.02237 (15)	
O1A	0.29396 (14)	0.97324 (11)	0.50941 (9)	0.0181 (4)	
O2A	0.23951 (14)	0.82784 (11)	0.56301 (8)	0.0186 (4)	
O3A	0.24928 (15)	0.89650 (11)	0.37882 (9)	0.0199 (4)	
O4A	0.36941 (15)	0.80282 (12)	0.45119 (9)	0.0196 (4)	
O5A	0.09197 (14)	0.92578 (12)	0.46412 (9)	0.0193 (4)	
O6A	0.14803 (15)	0.75007 (12)	0.45420 (9)	0.0220 (4)	
C1A	0.3082 (2)	0.97271 (16)	0.57562 (13)	0.0171 (5)	
C2A	0.3488 (2)	1.04342 (17)	0.61292 (13)	0.0208 (5)	
H2A	0.368091	1.099281	0.592823	0.025*	
C3A	0.3608 (2)	1.03143 (19)	0.68025 (14)	0.0278 (6)	
H3A	0.388467	1.079614	0.706268	0.033*	
C4A	0.3331 (3)	0.9502 (2)	0.70978 (15)	0.0293 (7)	
H4A	0.342127	0.943114	0.755821	0.035*	
C5A	0.2919 (2)	0.87832 (18)	0.67256 (14)	0.0246 (6)	
H5A	0.273006	0.822531	0.692909	0.030*	
C6A	0.2790 (2)	0.89005 (17)	0.60499 (13)	0.0169 (5)	
C7A	0.3287 (2)	0.85289 (16)	0.34636 (13)	0.0186 (5)	
C8A	0.3427 (2)	0.85377 (18)	0.27873 (13)	0.0249 (6)	
H8A	0.296315	0.887824	0.251048	0.030*	
C9A	0.4274 (2)	0.80286 (19)	0.25277 (14)	0.0270 (6)	
H9A	0.437837	0.801484	0.206588	0.032*	
C10A	0.4962 (2)	0.75445 (18)	0.29301 (14)	0.0269 (6)	
H10A	0.553816	0.721326	0.274072	0.032*	
C11A	0.4823 (2)	0.75350 (17)	0.36086 (14)	0.0230 (6)	
H11A	0.530330	0.721273	0.388597	0.028*	
C12A	0.3960 (2)	0.80109 (17)	0.38657 (13)	0.0179 (5)	
C13A	−0.0307 (2)	1.00875 (18)	0.37881 (14)	0.0231 (6)	
H13A	−0.094276	1.001230	0.350983	0.035*	
H13B	0.027660	1.035434	0.352935	0.035*	
H13C	−0.047955	1.048911	0.415678	0.035*	
C14A	−0.1055 (2)	0.86991 (17)	0.45376 (14)	0.0219 (6)	
H14A	−0.165463	0.861017	0.423232	0.033*	
H14B	−0.123587	0.918174	0.484975	0.033*	
H14C	−0.091925	0.813351	0.477709	0.033*	
C15A	0.1571 (3)	0.6507 (2)	0.34776 (16)	0.0470 (10)	
H15A	0.164735	0.588285	0.331680	0.070*	
H15B	0.209848	0.689715	0.325975	0.070*	
H15C	0.084493	0.672688	0.338069	0.070*	
C16A	0.0645 (2)	0.58991 (18)	0.45798 (15)	0.0267 (6)	
H16A	0.069675	0.528144	0.440385	0.040*	
H16B	−0.000313	0.619270	0.440775	0.040*	
H16C	0.060692	0.587406	0.506008	0.040*	
Ti1B	0.26978 (4)	0.36710 (3)	0.47110 (2)	0.01648 (10)	
S1B	0.48538 (6)	0.41289 (4)	0.39989 (3)	0.01951 (14)	
S2B	0.32921 (5)	0.15962 (4)	0.44898 (3)	0.01756 (14)	
O1B	0.22436 (15)	0.46008 (11)	0.53031 (9)	0.0204 (4)	
O2B	0.28384 (14)	0.30116 (11)	0.55326 (9)	0.0195 (4)	
O3B	0.22962 (15)	0.41888 (11)	0.38620 (9)	0.0190 (4)	
O4B	0.13334 (14)	0.30782 (12)	0.45810 (9)	0.0193 (4)	
O5B	0.41145 (15)	0.43189 (12)	0.45889 (9)	0.0213 (4)	
O6B	0.35320 (15)	0.26045 (11)	0.43304 (9)	0.0197 (4)	
C1B	0.2044 (2)	0.43151 (18)	0.59244 (13)	0.0190 (5)	
C2B	0.1564 (2)	0.48346 (19)	0.64125 (14)	0.0252 (6)	
H2B	0.137199	0.544949	0.633482	0.030*	
C3B	0.1374 (3)	0.4429 (2)	0.70182 (15)	0.0318 (7)	
H3B	0.105239	0.477313	0.736001	0.038*	
C4B	0.1645 (2)	0.3529 (2)	0.71306 (14)	0.0306 (7)	
H4B	0.148461	0.325989	0.754284	0.037*	
C5B	0.2149 (2)	0.30157 (19)	0.66489 (13)	0.0264 (6)	
H5B	0.235155	0.240446	0.673191	0.032*	
C6B	0.2353 (2)	0.34154 (17)	0.60413 (13)	0.0201 (5)	
C7B	0.1430 (2)	0.38045 (16)	0.35732 (13)	0.0189 (6)	
C8B	0.1074 (2)	0.39564 (18)	0.29362 (14)	0.0248 (6)	
H8B	0.145005	0.435848	0.265315	0.030*	
C9B	0.0150 (3)	0.35044 (19)	0.27199 (14)	0.0295 (6)	
H9B	−0.009738	0.360043	0.228406	0.035*	
C10B	−0.0408 (2)	0.29247 (18)	0.31242 (14)	0.0256 (6)	
H10B	−0.103731	0.263272	0.296679	0.031*	
C11B	−0.0054 (2)	0.27610 (17)	0.37675 (13)	0.0221 (6)	
H11B	−0.043914	0.236415	0.404958	0.027*	
C12B	0.0873 (2)	0.31931 (17)	0.39817 (13)	0.0176 (5)	
C13B	0.5285 (3)	0.52345 (18)	0.37659 (15)	0.0271 (7)	
H13D	0.584170	0.518513	0.342780	0.041*	
H13E	0.557799	0.555009	0.414997	0.041*	
H13F	0.467374	0.557725	0.359159	0.041*	
C14B	0.6051 (2)	0.37109 (19)	0.43729 (16)	0.0274 (7)	
H14D	0.659787	0.361118	0.403503	0.041*	
H14E	0.589689	0.313647	0.459669	0.041*	
H14F	0.631484	0.415538	0.469258	0.041*	
C15B	0.2614 (2)	0.12076 (17)	0.37805 (14)	0.0271 (6)	
H15D	0.250241	0.055054	0.381248	0.041*	
H15E	0.304702	0.134322	0.339095	0.041*	
H15F	0.191809	0.151379	0.374497	0.041*	
C16B	0.4557 (2)	0.10696 (18)	0.43641 (15)	0.0243 (6)	
H16D	0.447454	0.040889	0.439005	0.036*	
H16E	0.506156	0.127350	0.470277	0.036*	
H16F	0.483304	0.123567	0.392994	0.036*	

### Atomic displacement parameters (Å^2^)

**Table:**

	*U*^11^	*U*^22^	*U*^33^	*U*^12^	*U*^13^	*U*^23^
Ti1A	0.0163 (3)	0.0142 (2)	0.0187 (2)	0.00011 (18)	−0.0001 (2)	−0.00122 (16)
S1A	0.0179 (3)	0.0183 (3)	0.0196 (3)	0.0017 (2)	−0.0006 (3)	−0.0031 (2)
S2A	0.0197 (4)	0.0154 (3)	0.0319 (4)	−0.0009 (2)	0.0018 (3)	0.0002 (3)
O1A	0.0205 (10)	0.0161 (9)	0.0175 (9)	−0.0006 (7)	−0.0004 (7)	0.0008 (7)
O2A	0.0190 (10)	0.0166 (8)	0.0203 (9)	−0.0029 (7)	0.0006 (8)	−0.0004 (7)
O3A	0.0197 (10)	0.0204 (9)	0.0197 (9)	0.0040 (7)	−0.0002 (8)	0.0006 (7)
O4A	0.0210 (10)	0.0191 (9)	0.0188 (9)	0.0022 (7)	0.0007 (8)	0.0000 (7)
O5A	0.0159 (10)	0.0202 (9)	0.0218 (10)	0.0022 (7)	−0.0018 (8)	−0.0061 (8)
O6A	0.0213 (10)	0.0171 (9)	0.0276 (10)	−0.0012 (7)	−0.0001 (8)	−0.0050 (7)
C1A	0.0117 (13)	0.0177 (12)	0.0219 (13)	0.0041 (9)	0.0000 (10)	−0.0002 (10)
C2A	0.0173 (14)	0.0182 (12)	0.0271 (14)	−0.0019 (10)	−0.0020 (11)	−0.0010 (10)
C3A	0.0283 (16)	0.0273 (14)	0.0278 (15)	−0.0022 (11)	−0.0078 (13)	−0.0059 (12)
C4A	0.0307 (18)	0.0382 (17)	0.0190 (14)	−0.0020 (13)	−0.0057 (12)	−0.0028 (12)
C5A	0.0209 (15)	0.0271 (14)	0.0258 (15)	−0.0015 (11)	−0.0005 (12)	0.0052 (11)
C6A	0.0117 (13)	0.0180 (12)	0.0210 (13)	0.0007 (10)	−0.0013 (10)	−0.0019 (10)
C7A	0.0184 (15)	0.0159 (12)	0.0216 (13)	−0.0028 (10)	0.0029 (11)	−0.0005 (10)
C8A	0.0292 (17)	0.0238 (14)	0.0217 (14)	−0.0046 (11)	−0.0008 (12)	0.0023 (11)
C9A	0.0316 (17)	0.0285 (15)	0.0209 (15)	−0.0051 (12)	0.0087 (12)	−0.0040 (11)
C10A	0.0252 (15)	0.0231 (13)	0.0323 (16)	−0.0037 (12)	0.0075 (13)	−0.0041 (12)
C11A	0.0191 (15)	0.0178 (13)	0.0322 (15)	−0.0003 (11)	0.0035 (12)	−0.0019 (11)
C12A	0.0177 (14)	0.0158 (12)	0.0203 (14)	−0.0032 (10)	0.0017 (11)	−0.0010 (10)
C13A	0.0253 (16)	0.0236 (14)	0.0205 (15)	−0.0002 (11)	0.0004 (12)	0.0046 (11)
C14A	0.0199 (14)	0.0179 (13)	0.0280 (15)	0.0000 (10)	0.0008 (12)	0.0013 (11)
C15A	0.076 (3)	0.0343 (18)	0.0308 (18)	−0.0207 (17)	0.0158 (18)	−0.0095 (14)
C16A	0.0244 (15)	0.0186 (13)	0.0372 (17)	−0.0038 (11)	0.0053 (13)	−0.0019 (12)
Ti1B	0.0168 (2)	0.0141 (2)	0.0185 (2)	0.00012 (18)	0.0016 (2)	0.00020 (17)
S1B	0.0187 (3)	0.0180 (3)	0.0219 (3)	−0.0023 (3)	0.0019 (3)	−0.0018 (2)
S2B	0.0156 (3)	0.0140 (3)	0.0231 (3)	−0.0003 (2)	0.0016 (3)	0.0003 (2)
O1B	0.0216 (10)	0.0173 (8)	0.0224 (10)	0.0025 (7)	0.0018 (8)	0.0000 (7)
O2B	0.0204 (10)	0.0179 (9)	0.0202 (10)	0.0010 (7)	−0.0002 (8)	−0.0007 (7)
O3B	0.0204 (10)	0.0174 (8)	0.0193 (9)	−0.0031 (7)	0.0009 (8)	0.0014 (7)
O4B	0.0191 (10)	0.0207 (9)	0.0180 (9)	−0.0003 (7)	0.0006 (8)	0.0037 (7)
O5B	0.0206 (10)	0.0208 (10)	0.0226 (10)	−0.0010 (7)	0.0055 (9)	−0.0047 (8)
O6B	0.0205 (10)	0.0131 (8)	0.0256 (10)	−0.0024 (7)	0.0059 (8)	−0.0012 (7)
C1B	0.0168 (14)	0.0219 (13)	0.0182 (13)	−0.0019 (10)	0.0028 (10)	−0.0037 (10)
C2B	0.0211 (15)	0.0256 (14)	0.0289 (15)	0.0000 (11)	0.0003 (12)	−0.0067 (11)
C3B	0.0296 (18)	0.0444 (18)	0.0215 (15)	−0.0053 (14)	0.0052 (13)	−0.0102 (13)
C4B	0.0297 (17)	0.0444 (18)	0.0176 (14)	−0.0072 (13)	0.0008 (12)	−0.0036 (12)
C5B	0.0285 (16)	0.0293 (15)	0.0213 (14)	−0.0049 (11)	−0.0020 (12)	0.0026 (11)
C6B	0.0149 (13)	0.0254 (13)	0.0201 (13)	−0.0024 (10)	−0.0019 (11)	−0.0056 (11)
C7B	0.0215 (15)	0.0143 (12)	0.0210 (13)	0.0007 (10)	0.0011 (11)	−0.0016 (10)
C8B	0.0335 (17)	0.0217 (14)	0.0190 (14)	−0.0026 (11)	0.0032 (12)	0.0034 (11)
C9B	0.0386 (18)	0.0305 (15)	0.0195 (14)	−0.0009 (13)	−0.0054 (13)	−0.0029 (11)
C10B	0.0247 (16)	0.0260 (14)	0.0260 (15)	−0.0037 (11)	−0.0055 (12)	−0.0025 (11)
C11B	0.0211 (15)	0.0200 (13)	0.0252 (14)	−0.0020 (10)	0.0023 (12)	0.0002 (10)
C12B	0.0197 (14)	0.0164 (12)	0.0166 (13)	0.0030 (10)	0.0014 (11)	−0.0005 (10)
C13B	0.0288 (17)	0.0234 (14)	0.0290 (16)	−0.0050 (12)	−0.0016 (13)	0.0069 (12)
C14B	0.0231 (16)	0.0247 (15)	0.0344 (17)	0.0028 (11)	0.0018 (13)	0.0019 (13)
C15B	0.0273 (16)	0.0194 (13)	0.0347 (16)	−0.0006 (11)	−0.0076 (13)	−0.0025 (11)
C16B	0.0190 (15)	0.0219 (13)	0.0320 (16)	0.0024 (10)	0.0016 (12)	0.0026 (11)

### Geometric parameters (Å, º)

**Table:**

Ti1A—O4A	1.9113 (19)	Ti1B—O1B	1.9108 (18)
Ti1A—O1A	1.9220 (17)	Ti1B—O4B	1.9284 (19)
Ti1A—O2A	1.9346 (18)	Ti1B—O2B	1.9427 (18)
Ti1A—O3A	1.9564 (18)	Ti1B—O3B	1.9545 (18)
Ti1A—O6A	2.0414 (18)	Ti1B—O5B	2.0214 (19)
Ti1A—O5A	2.0416 (18)	Ti1B—O6B	2.0367 (17)
S1A—O5A	1.5509 (19)	S1B—O5B	1.5400 (19)
S1A—C14A	1.763 (3)	S1B—C13B	1.779 (3)
S1A—C13A	1.782 (3)	S1B—C14B	1.783 (3)
S2A—O6A	1.5364 (19)	S2B—O6B	1.5494 (18)
S2A—C16A	1.771 (3)	S2B—C15B	1.768 (3)
S2A—C15A	1.780 (3)	S2B—C16B	1.774 (3)
O1A—C1A	1.360 (3)	O1B—C1B	1.357 (3)
O2A—C6A	1.346 (3)	O2B—C6B	1.339 (3)
O3A—C7A	1.352 (3)	O3B—C7B	1.353 (3)
O4A—C12A	1.358 (3)	O4B—C12B	1.359 (3)
C1A—C2A	1.384 (4)	C1B—C2B	1.390 (4)
C1A—C6A	1.404 (3)	C1B—C6B	1.400 (4)
C2A—C3A	1.391 (4)	C2B—C3B	1.392 (4)
C2A—H2A	0.9500	C2B—H2B	0.9500
C3A—C4A	1.383 (4)	C3B—C4B	1.387 (4)
C3A—H3A	0.9500	C3B—H3B	0.9500
C4A—C5A	1.399 (4)	C4B—C5B	1.389 (4)
C4A—H4A	0.9500	C4B—H4B	0.9500
C5A—C6A	1.396 (4)	C5B—C6B	1.394 (4)
C5A—H5A	0.9500	C5B—H5B	0.9500
C7A—C8A	1.389 (4)	C7B—C8B	1.389 (4)
C7A—C12A	1.398 (4)	C7B—C12B	1.409 (4)
C8A—C9A	1.398 (4)	C8B—C9B	1.401 (4)
C8A—H8A	0.9500	C8B—H8B	0.9500
C9A—C10A	1.383 (4)	C9B—C10B	1.375 (4)
C9A—H9A	0.9500	C9B—H9B	0.9500
C10A—C11A	1.393 (4)	C10B—C11B	1.403 (4)
C10A—H10A	0.9500	C10B—H10B	0.9500
C11A—C12A	1.385 (4)	C11B—C12B	1.388 (4)
C11A—H11A	0.9500	C11B—H11B	0.9500
C13A—H13A	0.9800	C13B—H13D	0.9800
C13A—H13B	0.9800	C13B—H13E	0.9800
C13A—H13C	0.9800	C13B—H13F	0.9800
C14A—H14A	0.9800	C14B—H14D	0.9800
C14A—H14B	0.9800	C14B—H14E	0.9800
C14A—H14C	0.9800	C14B—H14F	0.9800
C15A—H15A	0.9800	C15B—H15D	0.9800
C15A—H15B	0.9800	C15B—H15E	0.9800
C15A—H15C	0.9800	C15B—H15F	0.9800
C16A—H16A	0.9800	C16B—H16D	0.9800
C16A—H16B	0.9800	C16B—H16E	0.9800
C16A—H16C	0.9800	C16B—H16F	0.9800
			
O4A—Ti1A—O1A	99.75 (8)	O1B—Ti1B—O4B	98.62 (8)
O4A—Ti1A—O2A	94.27 (8)	O1B—Ti1B—O2B	80.85 (8)
O1A—Ti1A—O2A	80.73 (7)	O4B—Ti1B—O2B	88.32 (8)
O4A—Ti1A—O3A	81.00 (8)	O1B—Ti1B—O3B	101.72 (8)
O1A—Ti1A—O3A	98.71 (8)	O4B—Ti1B—O3B	80.24 (8)
O2A—Ti1A—O3A	175.09 (8)	O2B—Ti1B—O3B	168.52 (8)
O4A—Ti1A—O6A	92.85 (8)	O1B—Ti1B—O5B	89.89 (8)
O1A—Ti1A—O6A	163.08 (8)	O4B—Ti1B—O5B	165.00 (8)
O2A—Ti1A—O6A	87.15 (7)	O2B—Ti1B—O5B	105.30 (8)
O3A—Ti1A—O6A	94.35 (8)	O3B—Ti1B—O5B	85.97 (8)
O4A—Ti1A—O5A	163.07 (8)	O1B—Ti1B—O6B	160.77 (8)
O1A—Ti1A—O5A	88.55 (7)	O4B—Ti1B—O6B	92.76 (8)
O2A—Ti1A—O5A	101.66 (8)	O2B—Ti1B—O6B	84.07 (7)
O3A—Ti1A—O5A	83.18 (8)	O3B—Ti1B—O6B	95.42 (8)
O6A—Ti1A—O5A	82.35 (7)	O5B—Ti1B—O6B	82.64 (7)
O5A—S1A—C14A	103.27 (12)	O5B—S1B—C13B	102.87 (13)
O5A—S1A—C13A	103.16 (12)	O5B—S1B—C14B	103.27 (13)
C14A—S1A—C13A	100.06 (13)	C13B—S1B—C14B	100.22 (14)
O6A—S2A—C16A	102.29 (12)	O6B—S2B—C15B	103.36 (12)
O6A—S2A—C15A	104.06 (14)	O6B—S2B—C16B	102.56 (12)
C16A—S2A—C15A	97.94 (16)	C15B—S2B—C16B	99.43 (14)
C1A—O1A—Ti1A	116.08 (15)	C1B—O1B—Ti1B	114.90 (15)
C6A—O2A—Ti1A	115.48 (15)	C6B—O2B—Ti1B	113.83 (15)
C7A—O3A—Ti1A	114.05 (15)	C7B—O3B—Ti1B	115.14 (15)
C12A—O4A—Ti1A	115.43 (16)	C12B—O4B—Ti1B	115.97 (16)
S1A—O5A—Ti1A	121.98 (10)	S1B—O5B—Ti1B	122.18 (11)
S2A—O6A—Ti1A	132.05 (11)	S2B—O6B—Ti1B	124.13 (11)
O1A—C1A—C2A	125.9 (2)	O1B—C1B—C2B	125.1 (2)
O1A—C1A—C6A	113.2 (2)	O1B—C1B—C6B	113.7 (2)
C2A—C1A—C6A	120.8 (2)	C2B—C1B—C6B	121.2 (2)
C1A—C2A—C3A	119.0 (2)	C1B—C2B—C3B	118.1 (3)
C1A—C2A—H2A	120.5	C1B—C2B—H2B	120.9
C3A—C2A—H2A	120.5	C3B—C2B—H2B	120.9
C4A—C3A—C2A	120.8 (3)	C4B—C3B—C2B	121.0 (3)
C4A—C3A—H3A	119.6	C4B—C3B—H3B	119.5
C2A—C3A—H3A	119.6	C2B—C3B—H3B	119.5
C3A—C4A—C5A	120.7 (3)	C3B—C4B—C5B	120.9 (3)
C3A—C4A—H4A	119.7	C3B—C4B—H4B	119.5
C5A—C4A—H4A	119.7	C5B—C4B—H4B	119.5
C6A—C5A—C4A	118.8 (3)	C4B—C5B—C6B	118.7 (3)
C6A—C5A—H5A	120.6	C4B—C5B—H5B	120.7
C4A—C5A—H5A	120.6	C6B—C5B—H5B	120.7
O2A—C6A—C5A	125.7 (2)	O2B—C6B—C5B	125.6 (2)
O2A—C6A—C1A	114.5 (2)	O2B—C6B—C1B	114.4 (2)
C5A—C6A—C1A	119.9 (2)	C5B—C6B—C1B	120.0 (2)
O3A—C7A—C8A	125.0 (2)	O3B—C7B—C8B	126.3 (2)
O3A—C7A—C12A	114.3 (2)	O3B—C7B—C12B	113.8 (2)
C8A—C7A—C12A	120.7 (3)	C8B—C7B—C12B	119.9 (2)
C7A—C8A—C9A	117.7 (3)	C7B—C8B—C9B	118.6 (2)
C7A—C8A—H8A	121.1	C7B—C8B—H8B	120.7
C9A—C8A—H8A	121.1	C9B—C8B—H8B	120.7
C10A—C9A—C8A	121.3 (3)	C10B—C9B—C8B	121.5 (3)
C10A—C9A—H9A	119.3	C10B—C9B—H9B	119.3
C8A—C9A—H9A	119.3	C8B—C9B—H9B	119.3
C9A—C10A—C11A	121.0 (3)	C9B—C10B—C11B	120.5 (3)
C9A—C10A—H10A	119.5	C9B—C10B—H10B	119.7
C11A—C10A—H10A	119.5	C11B—C10B—H10B	119.7
C12A—C11A—C10A	117.8 (3)	C12B—C11B—C10B	118.4 (2)
C12A—C11A—H11A	121.1	C12B—C11B—H11B	120.8
C10A—C11A—H11A	121.1	C10B—C11B—H11B	120.8
O4A—C12A—C11A	124.5 (2)	O4B—C12B—C11B	125.1 (2)
O4A—C12A—C7A	114.3 (2)	O4B—C12B—C7B	113.8 (2)
C11A—C12A—C7A	121.3 (3)	C11B—C12B—C7B	121.1 (2)
S1A—C13A—H13A	109.5	S1B—C13B—H13D	109.5
S1A—C13A—H13B	109.5	S1B—C13B—H13E	109.5
H13A—C13A—H13B	109.5	H13D—C13B—H13E	109.5
S1A—C13A—H13C	109.5	S1B—C13B—H13F	109.5
H13A—C13A—H13C	109.5	H13D—C13B—H13F	109.5
H13B—C13A—H13C	109.5	H13E—C13B—H13F	109.5
S1A—C14A—H14A	109.5	S1B—C14B—H14D	109.5
S1A—C14A—H14B	109.5	S1B—C14B—H14E	109.5
H14A—C14A—H14B	109.5	H14D—C14B—H14E	109.5
S1A—C14A—H14C	109.5	S1B—C14B—H14F	109.5
H14A—C14A—H14C	109.5	H14D—C14B—H14F	109.5
H14B—C14A—H14C	109.5	H14E—C14B—H14F	109.5
S2A—C15A—H15A	109.5	S2B—C15B—H15D	109.5
S2A—C15A—H15B	109.5	S2B—C15B—H15E	109.5
H15A—C15A—H15B	109.5	H15D—C15B—H15E	109.5
S2A—C15A—H15C	109.5	S2B—C15B—H15F	109.5
H15A—C15A—H15C	109.5	H15D—C15B—H15F	109.5
H15B—C15A—H15C	109.5	H15E—C15B—H15F	109.5
S2A—C16A—H16A	109.5	S2B—C16B—H16D	109.5
S2A—C16A—H16B	109.5	S2B—C16B—H16E	109.5
H16A—C16A—H16B	109.5	H16D—C16B—H16E	109.5
S2A—C16A—H16C	109.5	S2B—C16B—H16F	109.5
H16A—C16A—H16C	109.5	H16D—C16B—H16F	109.5
H16B—C16A—H16C	109.5	H16E—C16B—H16F	109.5
			
C14A—S1A—O5A—Ti1A	123.30 (13)	C13B—S1B—O5B—Ti1B	137.68 (14)
C13A—S1A—O5A—Ti1A	−132.84 (14)	C14B—S1B—O5B—Ti1B	−118.38 (14)
C16A—S2A—O6A—Ti1A	159.79 (16)	C15B—S2B—O6B—Ti1B	103.47 (15)
C15A—S2A—O6A—Ti1A	−98.66 (19)	C16B—S2B—O6B—Ti1B	−153.50 (14)
Ti1A—O1A—C1A—C2A	179.0 (2)	Ti1B—O1B—C1B—C2B	−170.4 (2)
Ti1A—O1A—C1A—C6A	0.2 (3)	Ti1B—O1B—C1B—C6B	8.6 (3)
O1A—C1A—C2A—C3A	−178.5 (3)	O1B—C1B—C2B—C3B	177.3 (3)
C6A—C1A—C2A—C3A	0.3 (4)	C6B—C1B—C2B—C3B	−1.6 (4)
C1A—C2A—C3A—C4A	0.1 (4)	C1B—C2B—C3B—C4B	−0.6 (4)
C2A—C3A—C4A—C5A	−0.2 (5)	C2B—C3B—C4B—C5B	2.2 (5)
C3A—C4A—C5A—C6A	−0.1 (4)	C3B—C4B—C5B—C6B	−1.7 (4)
Ti1A—O2A—C6A—C5A	−177.4 (2)	Ti1B—O2B—C6B—C5B	166.9 (2)
Ti1A—O2A—C6A—C1A	2.8 (3)	Ti1B—O2B—C6B—C1B	−13.3 (3)
C4A—C5A—C6A—O2A	−179.2 (3)	C4B—C5B—C6B—O2B	179.4 (3)
C4A—C5A—C6A—C1A	0.5 (4)	C4B—C5B—C6B—C1B	−0.4 (4)
O1A—C1A—C6A—O2A	−1.9 (3)	O1B—C1B—C6B—O2B	3.2 (3)
C2A—C1A—C6A—O2A	179.2 (2)	C2B—C1B—C6B—O2B	−177.7 (2)
O1A—C1A—C6A—C5A	178.3 (2)	O1B—C1B—C6B—C5B	−176.9 (2)
C2A—C1A—C6A—C5A	−0.6 (4)	C2B—C1B—C6B—C5B	2.1 (4)
Ti1A—O3A—C7A—C8A	169.6 (2)	Ti1B—O3B—C7B—C8B	−170.3 (2)
Ti1A—O3A—C7A—C12A	−7.5 (3)	Ti1B—O3B—C7B—C12B	9.2 (3)
O3A—C7A—C8A—C9A	−178.2 (2)	O3B—C7B—C8B—C9B	−179.3 (3)
C12A—C7A—C8A—C9A	−1.3 (4)	C12B—C7B—C8B—C9B	1.2 (4)
C7A—C8A—C9A—C10A	−1.1 (4)	C7B—C8B—C9B—C10B	0.4 (4)
C8A—C9A—C10A—C11A	1.1 (4)	C8B—C9B—C10B—C11B	−0.8 (4)
C9A—C10A—C11A—C12A	1.3 (4)	C9B—C10B—C11B—C12B	−0.4 (4)
Ti1A—O4A—C12A—C11A	−173.6 (2)	Ti1B—O4B—C12B—C11B	173.9 (2)
Ti1A—O4A—C12A—C7A	6.8 (3)	Ti1B—O4B—C12B—C7B	−5.8 (3)
C10A—C11A—C12A—O4A	176.7 (2)	C10B—C11B—C12B—O4B	−177.6 (2)
C10A—C11A—C12A—C7A	−3.7 (4)	C10B—C11B—C12B—C7B	2.0 (4)
O3A—C7A—C12A—O4A	0.6 (3)	O3B—C7B—C12B—O4B	−2.3 (3)
C8A—C7A—C12A—O4A	−176.6 (2)	C8B—C7B—C12B—O4B	177.2 (2)
O3A—C7A—C12A—C11A	−179.0 (2)	O3B—C7B—C12B—C11B	178.0 (2)
C8A—C7A—C12A—C11A	3.8 (4)	C8B—C7B—C12B—C11B	−2.4 (4)

### Hydrogen-bond geometry (Å, º)

**Table:**

*D*—H···*A*	*D*—H	H···*A*	*D*···*A*	*D*—H···*A*
C11*A*—H11*A*···O2*B*^i^	0.95	2.62	3.491 (3)	153
C13*A*—H13*C*···O5*A*^ii^	0.98	2.54	3.428 (3)	151
C14*A*—H14*B*···O1*A*^ii^	0.98	2.66	3.378 (3)	130
C14*A*—H14*B*···O5*A*^ii^	0.98	2.55	3.447 (3)	152
C14*A*—H14*C*···O4*B*^iii^	0.98	2.27	3.193 (3)	156
C13*B*—H13*E*···O5*B*^i^	0.98	2.60	3.495 (4)	151
C14*B*—H14*E*···O4*A*^i^	0.98	2.55	3.438 (3)	151
C14*B*—H14*F*···O1*B*^i^	0.98	2.56	3.336 (3)	136
C15*B*—H15*D*···O3*A*^iv^	0.98	2.34	3.307 (3)	171
C16*B*—H16*D*···O1*A*^iv^	0.98	2.59	3.186 (3)	119
C16*B*—H16*E*···O4*A*^i^	0.98	2.45	3.427 (3)	173

Symmetry codes: (i) −*x*+1, −*y*+1, −*z*+1; (ii) −*x*, −*y*+2, −*z*+1; (iii) −*x*, −*y*+1, −*z*+1; (iv) *x*, *y*−1, *z*.
